# An intelligent taekwondo coaching system based on augmented reality technology with real-time feedback mechanisms

**DOI:** 10.1038/s41598-025-24608-1

**Published:** 2025-11-19

**Authors:** Feng Yang, Zizhuo Wang

**Affiliations:** Institute of Physical Education, Dongshin University, Naju, 58245 South Korea

**Keywords:** Augmented reality, Motion recognition, Taekwondo training, Real-time feedback, Deep learning, Intelligent coaching, Engineering, Mathematics and computing

## Abstract

Traditional taekwondo training methods face limitations in providing objective, real-time feedback for technique improvement, relying primarily on subjective instructor observations that may lack precision and consistency. This research presents an innovative intelligent taekwondo coaching framework that integrates augmented reality technology with advanced motion analysis algorithms to deliver comprehensive, real-time training feedback. The system employs a modular architecture incorporating multi-modal sensor data acquisition, deep learning-based pose estimation, biomechanical analysis, and immersive AR visualization to create an interactive training environment. The motion recognition module utilizes convolutional neural networks specifically adapted for taekwondo techniques, achieving recognition accuracies exceeding 95% across nine fundamental technique categories with processing latencies below 25 milliseconds. The comprehensive evaluation framework assesses movement quality across eight dimensions including geometric accuracy, temporal coordination, and force generation, providing personalized feedback through AR overlays. Experimental validation with 47 practitioners across novice, intermediate, and advanced skill levels demonstrates significant improvements in learning efficiency and technique standardization compared to conventional training methods. User experience evaluation reveals high satisfaction ratings (average 8.5/10) across interface usability, feedback clarity, and learning effectiveness. The system successfully addresses critical gaps in martial arts instruction by democratizing access to high-quality technical guidance while maintaining the engagement and motivation essential for skill development. This research establishes important foundations for next-generation sports training technologies and provides valuable insights for developing similar intelligent coaching systems across diverse athletic disciplines.

## Introduction

Taekwondo, as one of the most practiced martial arts worldwide, demands precise technical execution and continuous refinement of movement patterns to achieve optimal performance outcomes^1^. The complexity of taekwondo techniques, encompassing dynamic kicking sequences, defensive maneuvers, and strategic positioning, necessitates comprehensive technical guidance that can provide immediate feedback on biomechanical accuracy and tactical effectiveness^2^. Traditional coaching methodologies, while foundational to martial arts instruction, face inherent limitations in delivering real-time, objective performance analysis that modern athletes require for accelerated skill development.

Conventional taekwondo training approaches primarily rely on visual observation and subjective assessment by human instructors, creating significant constraints in training effectiveness and consistency^3^. The temporal limitations of human perception restrict coaches’ ability to capture and analyze rapid movement sequences, while the subjective nature of traditional feedback mechanisms often lacks the precision necessary for identifying subtle technical deficiencies^4^. Furthermore, the instructor-to-student ratio in many training environments limits individualized attention, resulting in generic feedback that may not address specific biomechanical inadequacies or tactical weaknesses inherent to individual practitioners.

The emergence of augmented reality technology has demonstrated substantial potential for revolutionizing sports training methodologies across multiple disciplines^5^. AR systems enable the superimposition of digital information onto real-world environments, creating immersive training experiences that enhance spatial awareness and provide immediate visual feedback on performance parameters. Recent applications in sports training have demonstrated AR’s capacity to improve technique acquisition, reduce learning curves, and enhance athlete engagement through interactive visualization of complex movement patterns^6^.

Intelligent coaching systems represent a paradigm shift in sports training, leveraging artificial intelligence algorithms to provide personalized instruction and automated performance analysis^7^. These systems integrate multiple sensor modalities, computer vision techniques, and machine learning algorithms to create comprehensive training platforms that can adapt to individual learning styles and performance capabilities. The development of such systems in combat sports has shown promising results in technique recognition, movement analysis, and strategic planning, though their application in taekwondo remains relatively underexplored.

The integration of augmented reality technology with intelligent coaching systems presents unprecedented opportunities for enhancing taekwondo training effectiveness through real-time feedback mechanisms and comprehensive technical analysis. This research introduces a novel framework that combines AR visualization with advanced motion analysis algorithms to create an intelligent taekwondo coaching system capable of providing immediate, objective feedback on technique execution and tactical decision-making. The proposed system addresses critical gaps in existing training methodologies by offering precise biomechanical analysis, personalized instruction protocols, and immersive training experiences that enhance both technical proficiency and strategic understanding.

The innovation of this research lies in its comprehensive approach to intelligent coaching, incorporating real-time motion capture, biomechanical analysis, and AR-based feedback visualization into a unified training platform^8^. The system’s ability to provide immediate, objective feedback on technique execution represents a significant advancement over traditional training methods, while its adaptive learning capabilities ensure personalized instruction that evolves with individual practitioner development. The integration of tactical analysis components further distinguishes this framework by addressing both technical execution and strategic elements of taekwondo performance.

The significance of this research extends beyond technical innovation to encompass broader implications for martial arts education and athletic development. By democratizing access to high-quality technical instruction through intelligent coaching systems, this framework has the potential to standardize training quality across diverse educational settings and enhance the overall development of taekwondo practitioners worldwide.

This paper is structured to provide comprehensive coverage of the proposed intelligent taekwondo coaching framework, beginning with a detailed literature review of existing technologies and methodologies, followed by system architecture description, implementation details, experimental validation, and discussion of results and future research directions.

## Related technical research

### Augmented reality technology applications in sports training

Augmented Reality technology fundamentally operates by overlaying digital information onto real-world environments through sophisticated computer vision algorithms and display systems, creating interactive experiences that enhance user perception and engagement with their physical surroundings^9^. The core characteristics of AR systems include real-time processing capabilities, precise spatial registration of virtual objects, and seamless integration of digital content with physical environments, enabling users to interact with both virtual and real elements simultaneously. The technology’s ability to provide contextual information and immediate visual feedback makes it particularly suitable for educational and training applications where spatial awareness and real-time guidance are critical components of skill development.

Current research in AR applications for sports training has demonstrated significant progress across multiple athletic disciplines, with particular emphasis on technique refinement, performance analysis, and immersive training experiences^10^. Swimming stroke analysis systems have utilized AR to provide real-time feedback on body positioning and movement efficiency, while tennis training applications have incorporated AR visualization to enhance tactical understanding and shot placement accuracy. Basketball training systems have leveraged AR technology to create interactive shooting drills and defensive positioning exercises, demonstrating the technology’s versatility in addressing diverse training requirements across different sports modalities.

The analysis of existing AR sports application systems reveals both significant advantages and notable limitations that influence their practical implementation and effectiveness. Primary advantages include enhanced visual learning through immediate feedback mechanisms, improved spatial awareness through three-dimensional visualization, and increased training engagement through interactive and gamified experiences^11^. AR systems excel in providing objective performance metrics that supplement traditional subjective coaching observations, while their ability to create standardized training scenarios ensures consistent skill development across different training environments. However, current limitations include hardware constraints that affect system portability and user comfort, computational requirements that limit real-time processing capabilities, and accuracy issues in motion tracking that can compromise feedback reliability^12^.

The integration of AR technology with motion recognition systems has established several distinct application patterns that address different aspects of sports training and performance analysis. Overlay-based feedback systems represent the most common implementation, where AR interfaces display performance metrics, technique corrections, and instructional guidance directly within the athlete’s field of view during training sessions^13^. Template-based comparison systems utilize AR to superimpose ideal movement patterns onto real-time athlete performance, enabling immediate visual comparison between actual and target techniques. Predictive visualization systems employ AR to display projected movement trajectories and outcome scenarios, particularly valuable for strategic planning and tactical decision-making in competitive environments.

Advanced AR applications in sports training have begun incorporating machine learning algorithms to enhance motion recognition accuracy and provide personalized feedback based on individual performance characteristics^14^. These systems analyze movement patterns in real-time, identifying deviations from optimal technique and providing corrective guidance through AR visualization. The combination of computer vision techniques with AR rendering capabilities enables comprehensive biomechanical analysis that extends beyond simple motion tracking to include force analysis, timing assessment, and coordination evaluation. Such integrated approaches represent the current frontier in AR sports training applications, offering unprecedented levels of detail in performance analysis and instructional feedback.

The evolution of AR technology in sports training continues to address fundamental challenges related to system accuracy, user experience, and practical implementation in diverse training environments. Future developments focus on improving tracking precision, reducing computational latency, and enhancing the naturalistic integration of AR elements with physical training activities to create more effective and user-friendly training solutions.

### Human motion recognition and analysis algorithms

Deep learning-based human pose estimation methods have revolutionized the field of motion analysis by providing robust and accurate joint localization capabilities that form the foundation for comprehensive movement assessment^15^. Convolutional neural networks, particularly those implementing multi-stage refinement architectures, demonstrate superior performance in detecting and tracking human skeletal keypoints across diverse motion scenarios. The pose estimation process typically involves the application of heatmap regression techniques for initial 2D joint detection, where the probability distribution of joint locations is computed using the following formulation:

1 $$\:P\left({x}_{i},{y}_{i}\right)=\frac{1}{2\pi\:{\sigma\:}^{2}}\text{e}\text{x}\text{p}\left(-\frac{{\left(x-{x}_{i}\right)}^{2}+{\left(y-{y}_{i}\right)}^{2}}{2{\sigma\:}^{2}}\right)$$

For 3D pose estimation required by the AR system, this 2D detection is extended to three-dimensional space by incorporating depth information from RGB-D sensors, where the 3D probability distribution is formulated as:

2 $$\:P\left({x}_{i},{y}_{i},{z}_{i}\right)=\frac{1}{{\left(2\pi\:\right)}^{3/2}{\sigma\:}^{3}}\text{e}\text{x}\text{p}\left(-\frac{{\left(x-{x}_{i}\right)}^{2}+{\left(y-{y}_{i}\right)}^{2}+{\left(z-{z}_{i}\right)}^{2}}{2{\sigma\:}^{2}}\right)$$

where $\:\left({x}_{i},{y}_{i}\right)$ represents the coordinates of the i-th joint, and $\:\sigma\:$ controls the spatial spread of the probability distribution^16^. Advanced pose estimation frameworks incorporate temporal consistency constraints to enhance tracking stability and reduce jitter in dynamic movement sequences, particularly crucial for martial arts applications where rapid transitions between techniques occur frequently.

Motion trajectory tracking algorithms extend beyond static pose estimation to capture the dynamic characteristics of human movement through temporal analysis of joint positions and velocities^17^. Kalman filtering approaches have proven effective for predicting future joint positions based on current motion states, incorporating both positional and velocity information to maintain tracking continuity during occlusions or rapid movements. The state prediction model for joint tracking can be expressed as:

3 $$\:{\mathbf{x}}_{k+1}=\mathbf{A}{\mathbf{x}}_{k}+\mathbf{B}{\mathbf{u}}_{k}+{\mathbf{w}}_{k}$$

where $\:{\mathbf{x}}_{k}$ represents the joint state vector at time k, $\:\mathbf{A}$ is the state transition matrix, $\:\mathbf{B}$ is the control input matrix, and $\:{\mathbf{w}}_{k}$ represents process noise^18^. Particle filter implementations have demonstrated superior performance in handling non-linear motion dynamics and multi-modal probability distributions that characterize complex martial arts movements.

Feature extraction methods for taekwondo techniques require specialized approaches that capture the unique biomechanical characteristics of kicking motions, defensive maneuvers, and transitional movements^19^. Geometric features derived from joint angle computations provide fundamental information about limb positioning and movement patterns, while kinematic features extracted from velocity and acceleration profiles reveal the dynamic aspects of technique execution. The angular velocity feature for joint rotations is computed using:

4 $$\:{\omega\:}_{j}\left(t\right)=\frac{d{\theta\:}_{j}\left(t\right)}{dt}=\frac{{\theta\:}_{j}\left(t\right)-{\theta\:}_{j}\left(t-1\right)}{\varDelta\:t}$$

where $\:{\theta\:}_{j}\left(t\right)$ represents the joint angle at time t, and $\:\varDelta\:t$ is the temporal sampling interval^20^. Frequency domain analysis through Fourier transform techniques enables the identification of periodic motion patterns and rhythm characteristics that distinguish skilled performances from novice executions.

Mathematical models for motion quality assessment integrate multiple biomechanical parameters to generate comprehensive performance scores that reflect technique proficiency and execution standards^21^. The quality assessment framework typically employs weighted combination of individual feature scores, where the overall quality metric is defined as:

5 $$\:Q=\sum\limits_{i=1}^{n}{w}_{i}\cdot\:{f}_{i}\cdot\:{e}^{-{\lambda\:}_{i}\cdot\:{d}_{i}}$$

where $\:{w}_{i}$ represents the weight for feature i, $\:{f}_{i}$ is the normalized feature value, $\:{d}_{i}$ is the deviation from the reference template, and $\:{\lambda\:}_{i}$ controls the penalty for deviations^22^. This formulation enables the incorporation of expert knowledge through weight assignment while maintaining objective assessment criteria based on measurable biomechanical parameters. Advanced quality assessment models incorporate machine learning techniques to automatically optimize weight parameters and threshold values based on training data from expert demonstrations, enhancing the system’s ability to provide accurate and consistent performance evaluations across diverse skill levels and technique variations.

### Real-time feedback system design theory

Real-time feedback systems for sports training applications must satisfy stringent performance requirements that ensure immediate response capabilities and consistent system behavior under varying computational loads^23^. The fundamental design principles for real-time systems emphasize deterministic response times, where system latency must remain within acceptable bounds to maintain the effectiveness of feedback mechanisms. Critical performance metrics include end-to-end latency, which encompasses data acquisition, processing, and output generation phases, with typical requirements demanding response times below 100 milliseconds for optimal user experience in interactive training scenarios. System throughput requirements must accommodate continuous data streams from multiple sensor modalities while maintaining processing accuracy, necessitating careful resource allocation and algorithmic optimization to prevent performance degradation during peak operational periods.

The psychological foundation of feedback mechanisms in motor learning establishes that effective feedback systems must align with cognitive processing capabilities and learning theory principles to maximize skill acquisition and retention^24^. Knowledge of results and knowledge of performance represent two fundamental feedback categories that serve distinct roles in motor skill development, where knowledge of results provides information about movement outcomes while knowledge of performance focuses on movement execution characteristics. The timing of feedback delivery significantly influences learning effectiveness, with immediate feedback promoting rapid error correction but potentially creating dependency, while delayed feedback encourages internal error detection mechanisms and promotes long-term skill retention. Augmented feedback systems must balance between providing sufficient information for skill improvement and maintaining learner autonomy to develop internal feedback capabilities essential for independent performance optimization.

Multi-modal interaction interface design methods integrate visual, auditory, and haptic feedback channels to create comprehensive training experiences that accommodate diverse learning preferences and enhance information processing efficiency^25^. Visual feedback interfaces typically employ color-coded indicators, trajectory overlays, and performance metrics displays that provide immediate visual cues about movement quality and execution accuracy. Auditory feedback systems utilize tone variations, rhythm patterns, and verbal instructions to convey temporal information and provide guidance during movement execution when visual attention is directed elsewhere. Haptic feedback mechanisms incorporate vibrotactile stimulation and force feedback to provide tactile cues about movement timing, intensity, and directional corrections, particularly valuable for kinesthetic learners who benefit from physical sensations during skill acquisition.

The integration of multiple feedback modalities requires careful consideration of information hierarchy and channel capacity limitations to prevent cognitive overload and ensure effective information processing^26^. Design principles emphasize complementary rather than redundant information presentation across modalities, where each channel provides unique information that enhances overall system effectiveness without creating conflicting or overwhelming stimuli. Adaptive feedback systems adjust information presentation based on user performance levels and learning progress, providing detailed guidance for novice users while reducing feedback complexity for advanced practitioners who require minimal external guidance.

System usability evaluation theoretical frameworks establish standardized methodologies for assessing the effectiveness, efficiency, and satisfaction aspects of real-time feedback systems in training environments^27^. The Technology Acceptance Model provides a structured approach for evaluating user acceptance factors, including perceived usefulness, perceived ease of use, and behavioral intention to adopt the technology. Usability metrics encompass task completion rates, error frequencies, learning curve characteristics, and subjective satisfaction ratings that collectively indicate system effectiveness in achieving training objectives. Cognitive load assessment frameworks evaluate the mental effort required to process feedback information, ensuring that system complexity does not exceed user processing capabilities and interfere with motor skill development.

Longitudinal usability studies examine system effectiveness over extended training periods, assessing factors such as user engagement maintenance, skill transfer to real-world scenarios, and long-term learning outcomes^28^. These evaluation frameworks incorporate both quantitative performance metrics and qualitative user experience assessments to provide comprehensive understanding of system impact on training effectiveness and user satisfaction, enabling continuous system refinement and optimization based on empirical evidence.

## System design and implementation

### Overall system architecture design

The intelligent taekwondo coaching system employs a modular architecture that integrates augmented reality visualization, real-time motion analysis, and intelligent feedback mechanisms into a cohesive training platform designed to enhance technique acquisition and performance optimization^29^. The system architecture follows a layered design approach that separates data acquisition, processing, analysis, and presentation functionalities, enabling scalable development and maintenance while ensuring optimal performance across diverse hardware configurations.

The overall framework consists of five primary functional layers, each with specific functional responsibilities and data flow characteristics:

#### Sensor data acquisition layer:

Manages multi-modal sensor inputs including RGB-D cameras, IMU sensors, and pressure sensors, with synchronized data collection at 30 FPS for visual data and 1000 Hz for kinematic data. This layer outputs standardized sensor data streams to the motion processing layer.

#### Motion processing layer:

Performs real-time 3D pose estimation by combining 2D joint detection from Eq. (1) with depth data using Eq. (2), generating 25-point skeletal representations at 30 FPS with sub-millimeter spatial accuracy.

#### Intelligent analysis layer:

Evaluates technique quality through multi-dimensional assessment algorithms, comparing observed movements against expert templates and generating performance scores across eight evaluation dimensions with processing latencies below 15 milliseconds.

#### Augmented reality rendering layer: 

Creates immersive feedback visualization by overlaying virtual coaching elements onto real environments, maintaining 60 FPS rendering performance with accurate spatial registration using visual-inertial tracking algorithms.

#### User interface layer:

Provides intuitive interaction mechanisms through gesture recognition and voice commands, enabling real-time training parameter adjustment and personalized feedback intensity control.

**Fig. 1:**
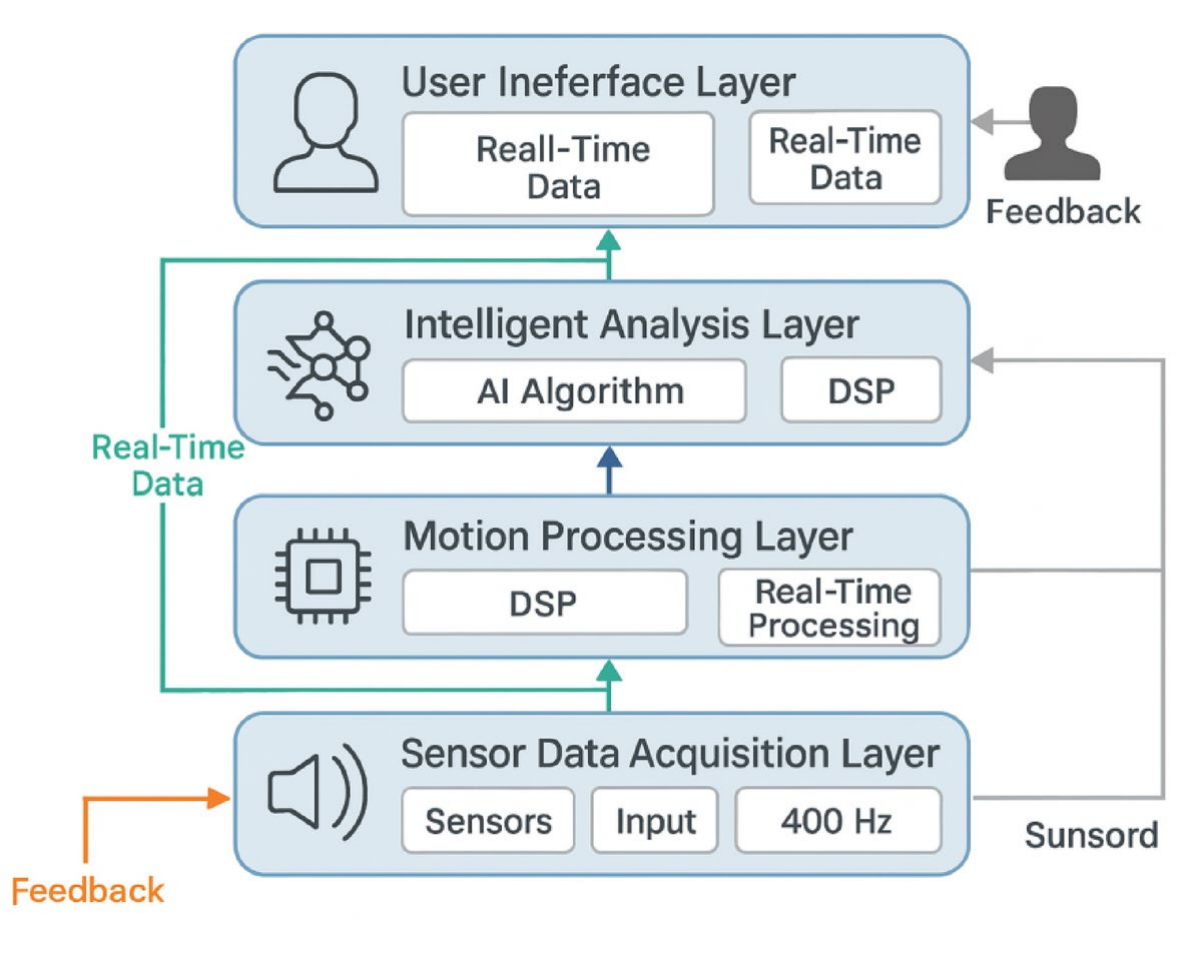
Intelligent Taekwondo Coaching System Architecture Overview. The diagram illustrates the modular design with data flow between sensor acquisition, motion processing, intelligent analysis, AR rendering, and user interface layers, demonstrating the system’s real-time feedback generation capabilities.

As illustrated in Fig. 1, the system architecture demonstrates the interconnected relationships between functional modules and the bidirectional data flow that enables real-time feedback generation and adaptive learning capabilities. The sensor data acquisition layer interfaces with multiple input devices to capture comprehensive movement information, while the motion processing layer performs real-time pose estimation and trajectory tracking. The intelligent analysis layer evaluates technique quality and generates performance assessments, which are subsequently visualized through the augmented reality rendering layer and presented via the user interface layer.

The sensor data acquisition module serves as the primary interface between the physical training environment and the digital analysis system, incorporating multiple sensing modalities to capture comprehensive movement data^30^. This module manages RGB-D camera inputs for visual motion capture, inertial measurement unit data for kinematic analysis, and pressure sensor information for force distribution assessment. The data synchronization component within this module ensures temporal alignment of multi-modal sensor streams, implementing hardware-level timestamping and software-based interpolation to maintain data integrity across different sampling rates and latency characteristics.

The motion processing module implements advanced computer vision algorithms to extract skeletal pose information and track movement trajectories in real-time, utilizing optimized deep learning models specifically adapted for martial arts movement recognition^31^. The 3D pose estimation process begins with 2D joint detection using Eq. (1) applied to RGB image data, followed by depth fusion from the RGB-D sensor to generate 3D coordinates. For each detected 2D joint $\:\left({x}_{i},{y}_{i}\right)$, the corresponding depth value $\:{d}_{i}$ is extracted from the aligned depth map, and the 3D position is computed as:

6 $$\:\begin{array}{lll}{X}_{i}&\:=\left({x}_{i}-{c}_{x}\right)\times\:{d}_{i}/{f}_{x}\\\:{Y}_{i}&\:=\left({y}_{i}-{c}_{y}\right)\times\:{d}_{i}/{f}_{y}\\\:{Z}_{i}&\:={d}_{i}\end{array}\:$$

where $\:\left({c}_{x},{c}_{y}\right)$ represents the camera optical center and $\:\left({f}_{x},{f}_{y}\right)$ are the focal lengths in pixel units.

The pose estimation component employs convolutional neural network architectures to identify joint locations and orientations, while the trajectory tracking component maintains temporal consistency through Kalman filtering and particle filter implementations. Feature extraction algorithms within this module compute geometric, kinematic, and dynamic parameters that characterize taekwondo techniques, generating standardized movement descriptors for subsequent analysis phases.

The intelligent analysis module represents the core computational component that evaluates technique quality, identifies performance deviations, and generates personalized feedback recommendations based on comparative analysis with expert movement templates^32^. This module integrates machine learning classifiers for technique recognition, statistical analysis tools for performance assessment, and rule-based systems for feedback generation. The adaptive learning component continuously refines analysis parameters based on user performance history and training progress, enabling personalized instruction that evolves with individual skill development.

The hardware configuration requirements for optimal system performance encompass specialized sensing equipment, high-performance computing components, and augmented reality display devices that collectively enable real-time processing and visualization capabilities. Table 1 provides comprehensive specifications for the essential hardware components, detailing the technical requirements and functional roles of each device type within the system architecture.

**Table 1:** Hardware configuration requirements for intelligent taekwondo coaching System.

Device Type	Specification Parameters	Quantity	Functional Description
RGB-D Camera	Intel RealSense D435i, 1920 × 1080@30fps, depth range 0.3–3 m	3	Multi-angle motion capture and depth perception for 3D pose estimation
IMU Sensors	MPU-9250, 9-axis motion tracking, 1000 Hz sampling rate	8	High-frequency kinematic data collection for limb movement analysis
Processing Unit	NVIDIA RTX 4080, 32GB RAM, Intel i9-12900 K	1	Real-time deep learning inference and parallel data processing
AR Headset	Microsoft HoloLens 2, 2 K display per eye, 43° diagonal FOV, spatial tracking, 4-hour battery life	1	Immersive feedback visualization with limited field of view requiring careful content positioning
Pressure Sensors	FlexiForce A201, 0–445 N force range, 200 Hz response	4	Ground reaction force measurement for balance and power analysis
Network Router	WiFi 6 standard, 1Gbps throughput, low latency	1	High-speed data transmission and sensor synchronization

The data flow processing mechanism implements a pipeline architecture that ensures efficient information transfer between system components while maintaining real-time performance requirements^33^. Raw sensor data undergoes preprocessing stages including noise reduction, calibration correction, and format standardization before entering the motion analysis pipeline. The processed movement data flows through feature extraction algorithms, quality assessment models, and feedback generation systems, with intermediate results cached to enable responsive user interaction and system adaptability.

The software development environment integrates multiple frameworks and libraries to support cross-platform compatibility and efficient algorithm implementation, utilizing Python for machine learning components, C + + for real-time processing modules, and Unity3D for augmented reality visualization development. The modular software architecture enables independent development and testing of individual components while maintaining system integration through standardized communication protocols and data exchange formats. Version control systems and continuous integration pipelines ensure software quality and facilitate collaborative development across distributed research teams.

### Augmented reality rendering and tracking algorithms

**Fig. 2:**
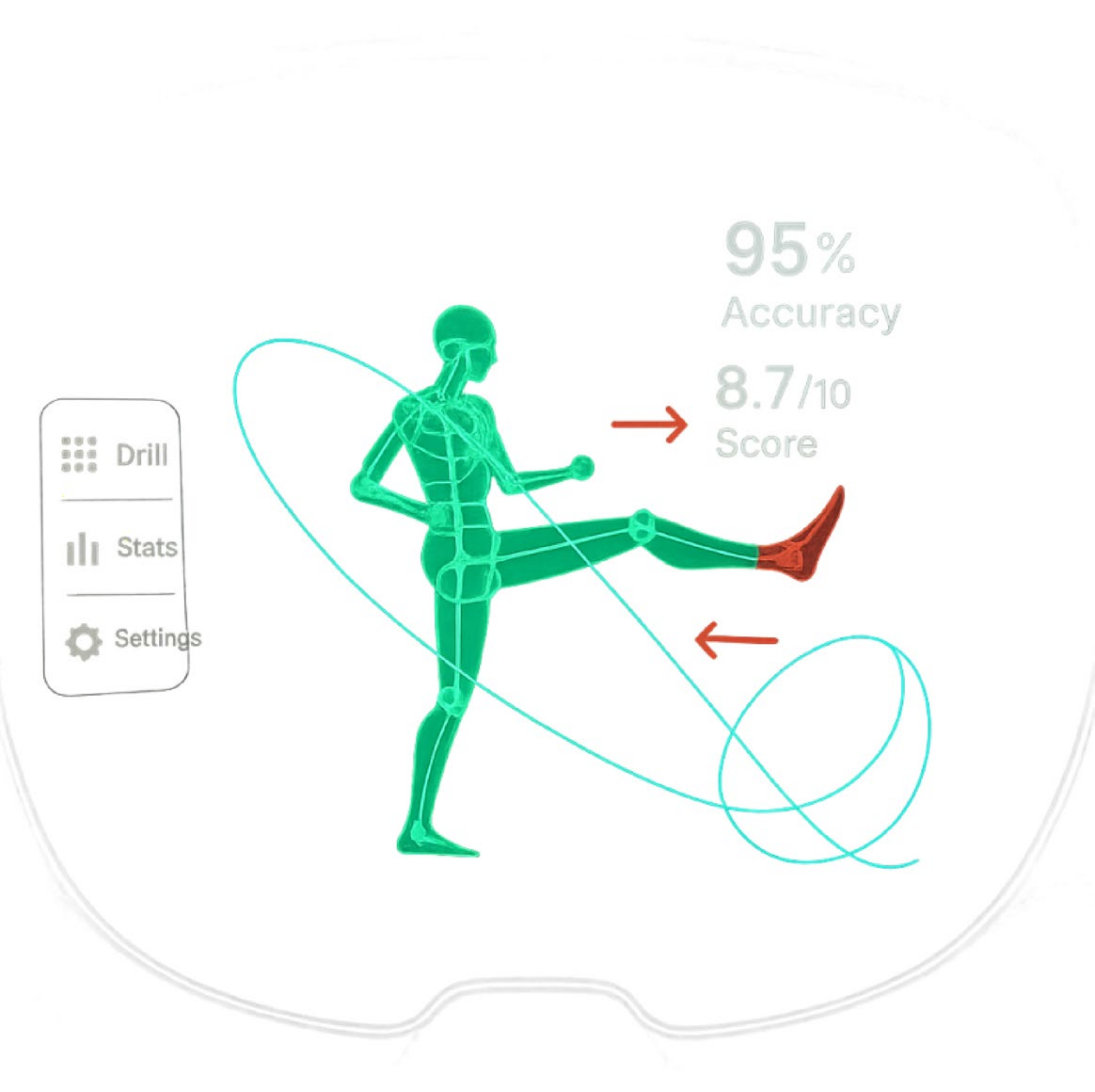
AR interface implementation for taekwondo training system.

The core algorithms for AR scene rendering in the intelligent taekwondo coaching system implement a multi-stage pipeline that transforms three-dimensional virtual coaching elements into seamlessly integrated visual overlays within the real training environment^34^. The system displays multiple types of virtual content through the HoloLens 2 interface, including real-time technique trajectories rendered as colored 3D paths, expert demonstration templates displayed as semi-transparent skeletal overlays, performance scores shown as floating numerical indicators, and corrective guidance arrows highlighting movement adjustments needed for technique improvement. As illustrated in Fig. 2, these AR elements are spatially registered within the user’s field of view to provide intuitive visual feedback without obstructing natural movement patterns.

The rendering pipeline employs physically-based rendering techniques that calculate lighting interactions, material properties, and geometric transformations to achieve photorealistic virtual object representation. The scene graph management system organizes virtual coaching elements hierarchically, enabling efficient culling operations and level-of-detail optimizations that maintain consistent frame rates during complex training scenarios involving multiple simultaneous feedback visualizations.

Three-dimensional spatial positioning and tracking methods form the foundation of accurate AR overlay registration, utilizing simultaneous localization and mapping algorithms combined with inertial sensor fusion to establish robust coordinate system relationships between virtual and physical spaces^35^. The system implements a hybrid tracking approach that combines visual-inertial odometry with marker-based registration to achieve millimeter-level positioning accuracy essential for precise technique feedback visualization. Keypoint detection algorithms identify distinctive features within the training environment, while bundle adjustment optimization refines camera pose estimates through iterative minimization of reprojection errors across multiple video frames.

**Fig. 3:**
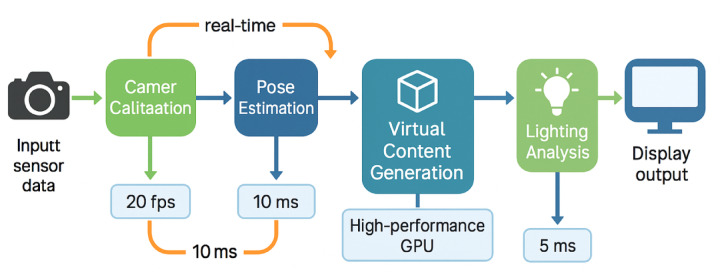
Augmented reality rendering pipeline for taekwondo training system. the diagram depicts the sequential processing stages from camera input and sensor data through pose estimation, virtual object generation, lighting matching, and final composite rendering, demonstrating the comprehensive workflow required for real-time ar feedback visualization.

The complete AR rendering workflow, as demonstrated in Fig. 3, illustrates the comprehensive processing pipeline from sensor input acquisition through final display output generation. The pipeline integrates camera calibration, pose estimation, object tracking, virtual content generation, lighting analysis, and composite rendering stages to produce coherent augmented reality visualizations that enhance training effectiveness without compromising visual fidelity.

Virtual object fusion with real environments requires sophisticated occlusion handling and depth testing algorithms that ensure virtual coaching elements appear naturally integrated within the physical training space^36^. The depth buffer management system utilizes RGB-D camera data to construct accurate three-dimensional scene representations, enabling proper occlusion relationships between virtual feedback elements and real-world objects such as training equipment or practitioners. Alpha blending techniques implement transparency effects for virtual trajectories and performance indicators, while z-buffering algorithms ensure correct depth ordering when multiple virtual elements overlap within the augmented scene.

Performance comparison analysis of various tracking algorithms reveals significant differences in accuracy, computational efficiency, and robustness characteristics that influence system implementation decisions. Table 2 presents comprehensive performance metrics for leading tracking approaches, highlighting the trade-offs between precision and computational requirements that guide algorithm selection for real-time training applications.

**Table 2:** Performance comparison of tracking algorithms for AR implementation.

Algorithm Name	Accuracy (mm)	Speed (FPS)	Robustness Score	Memory Usage (MB)	Application Scenario
ORB-SLAM3	2.1	30	8.5/10	145	Dynamic environment tracking
Visual-Inertial	1.8	45	9.2/10	89	High-motion scenarios
AprilTag Markers	0.9	60	7.8/10	32	Controlled environment
ArUco Detection	1.2	55	8.1/10	28	Structured training spaces
PTAM Enhanced	2.5	25	7.2/10	167	Feature-rich environments

The comparative analysis presented in Table 2 demonstrates that visual-inertial tracking algorithms provide optimal balance between accuracy and robustness for martial arts training applications, while marker-based approaches offer superior precision in controlled training environments where environmental modification is feasible.

Lighting matching and shadow processing algorithms ensure visual coherence between virtual coaching elements and the real training environment through real-time illumination analysis and synthetic shadow generation^37^. The illumination estimation component analyzes ambient and directional lighting characteristics within the training space using spherical harmonics decomposition of environment maps captured through omnidirectional cameras. Shadow mapping techniques generate realistic cast shadows from virtual objects onto real surfaces, utilizing depth texture sampling and percentage-closer filtering to achieve soft shadow edges that match natural lighting conditions.

Advanced lighting models incorporate physically-based material properties for virtual coaching equipment and feedback indicators, implementing bidirectional reflectance distribution functions that accurately simulate light interaction with synthetic surfaces^38^. The shadow rendering pipeline employs cascaded shadow mapping for directional lights and point light shadow mapping for localized illumination sources, ensuring consistent visual integration across diverse training environments with varying lighting conditions.

Real-time performance optimization strategies include temporal reprojection techniques that reuse rendering results from previous frames to reduce computational overhead while maintaining visual quality^39^. Level-of-detail systems automatically adjust virtual object complexity based on viewing distance and rendering performance requirements, while frustum culling algorithms eliminate unnecessary rendering operations for virtual elements outside the current field of view. These optimization techniques collectively enable sustained real-time performance on mobile AR platforms while preserving the visual fidelity necessary for effective training feedback delivery.

The integration of machine learning-based tracking enhancement algorithms provides adaptive performance optimization that learns from environmental characteristics and user movement patterns to predict optimal tracking parameters^40^. These adaptive systems continuously monitor tracking accuracy metrics and automatically adjust algorithm parameters to maintain robust performance across diverse training scenarios and environmental conditions, ensuring consistent AR overlay registration throughout extended training sessions.

### Motion recognition and evaluation module

The establishment of a comprehensive taekwondo standard motion database forms the foundational component for accurate technique recognition and assessment, incorporating kinematic and biomechanical data from expert demonstrations across all fundamental techniques and their variations^41^. The database construction process involves systematic motion capture of standardized techniques including basic kicks, blocks, strikes, and stances, with each technique recorded from multiple viewing angles and execution speeds to ensure comprehensive coverage of movement variations. The data preprocessing pipeline normalizes temporal sequences and spatial coordinates to create standardized reference templates, implementing time-warping algorithms to account for natural variation in execution timing while preserving essential movement characteristics.

Convolutional neural network architectures specifically designed for temporal sequence analysis provide robust motion classification capabilities that distinguish between different taekwondo techniques with high accuracy and computational efficiency^42^. The network architecture employs a hybrid approach combining spatial feature extraction through 2D convolutions applied to pose sequence data and temporal pattern recognition through 1D convolutions across time dimensions. The classification probability for technique i is computed using the softmax function:

7 $$\:P\left({c}_{i}|x\right)=\frac{{e}^{{z}_{i}}}{\sum_{j=1}^{N}{e}^{{z}_{j}}}$$

where $\:{z}_{i}$ represents the network output for class i, and N is the total number of technique classes. The loss function for training incorporates cross-entropy with regularization terms to prevent overfitting:

8 $$\:L=-\frac{1}{m}\sum\limits_{j=1}^{m}\sum\limits_{i=1}^{N}{y}_{j,i}\text{l}\text{o}\text{g}\left(P\left({c}_{i}|{x}_{j}\right)\right)+\lambda\:\parallel\:\theta\:{\parallel\:}^{2}$$

where m is the batch size, $\:{y}_{j,i}$ is the ground truth label, and $\:\lambda\:$ controls regularization strength.

Quantitative evaluation methods for motion completion and standardization employ multi-dimensional assessment frameworks that analyze geometric accuracy, temporal characteristics, and dynamic properties of technique execution^43^. The geometric accuracy component evaluates joint angle deviations from reference templates using angular distance metrics:

9 $$\:{D}_{angle}=\sqrt{\frac{1}{J}\sum\limits_{j=1}^{J}{\left({\theta\:}_{j}-{\theta\:}_{ref,j}\right)}^{2}}$$

where J represents the number of joints and $\:{\theta\:}_{ref,j}$ is the reference angle for joint j. Temporal evaluation metrics assess movement timing and rhythm characteristics through correlation analysis between observed and reference velocity profiles:

10 $$\:{C}_{temporal}=\frac{\sum\nolimits_{t=1}^{T}\left({v}_{t}-\stackrel{-}{v}\right)\left({v}_{ref,t}-{\stackrel{-}{v}}_{ref}\right)}{\sqrt{\sum\nolimits_{t=1}^{T}{\left({v}_{t}-\stackrel{-}{v}\right)}^{2}}\sqrt{\sum\nolimits_{t=1}^{T}{\left({v}_{ref,t}-{\stackrel{-}{v}}_{ref}\right)}^{2}}}$$

where $\:{v}_{t}$ represents velocity at time t and $\:\stackrel{-}{v}$ denotes mean velocity.

The comprehensive evaluation framework incorporates multiple assessment dimensions with weighted scoring mechanisms that reflect the relative importance of different technique aspects. As detailed in Table 3, the evaluation system encompasses geometric accuracy, temporal coordination, force generation, balance maintenance, technique fluidity, spatial awareness, energy efficiency, and tactical appropriateness, each contributing specific feedback information for technique improvement.

**Table 3:** Motion evaluation index system for Taekwondo technique Assessment.

Evaluation Dimension	Weight	Scoring Standard	Threshold Range	Feedback Type
Geometric Accuracy	0.25	Joint angle deviation from template	0–15° excellent, 15–30° good	Corrective positioning
Temporal Coordination	0.20	Timing sequence accuracy	> 0.8 correlation excellent	Rhythm adjustment
Force Generation	0.15	Peak velocity and acceleration	> 85% reference velocity	Power enhancement
Balance Maintenance	0.15	Center of mass stability	< 5 cm displacement excellent	Stability training
Technique Fluidity	0.10	Movement smoothness index	> 0.9 smoothness excellent	Flow optimization
Spatial Awareness	0.08	Distance and positioning accuracy	< 10 cm error excellent	Spatial correction
Energy Efficiency	0.05	Movement economy ratio	> 0.85 efficiency excellent	Economy guidance
Tactical Appropriateness	0.02	Context-specific execution	Expert evaluation scale	Strategic advice

The multi-dimensional assessment framework presented in Table 3 enables comprehensive technique evaluation that addresses both fundamental movement mechanics and advanced performance characteristics, providing detailed feedback across all critical aspects of taekwondo execution.

Dynamic quality scoring combines individual dimension assessments through weighted aggregation with adaptive threshold adjustment based on practitioner skill level:

11 $$\:{Q}_{total}=\sum\limits_{i=1}^{D}{w}_{i}\cdot\:\frac{{S}_{i}-{S}_{min,i}}{{S}_{max,i}-{S}_{min,i}}\cdot\:{e}^{-{\alpha\:}_{i}\cdot\:\left|{S}_{i}-{S}_{target,i}\right|}$$

where $\:{w}_{i}$ represents dimension weights, $\:{S}_{i}$ is the score for dimension i, and $\:{\alpha\:}_{i}$ controls penalty sensitivity for deviations from target performance levels^44^.

Personalized training recommendation generation mechanisms analyze individual performance patterns and learning progress to create customized training protocols that address specific weaknesses and build upon existing strengths^45^. The recommendation engine employs collaborative filtering techniques combined with content-based analysis to identify optimal training sequences and difficulty progressions. The recommendation score for training exercise j for user u is calculated as:

12 $$\:{R}_{u,j}={\stackrel{-}{r}}_{u}+\frac{\sum\:_{i\in\:N\left(u\right)}\text{sim}\left(u,i\right)\cdot\:\left({r}_{i,j}-{\stackrel{-}{r}}_{i}\right)}{\sum\:_{i\in\:N\left(u\right)}\left|\text{sim}\left(u,i\right)\right|}$$

where $\:{\stackrel{-}{r}}_{u}$ is the average rating for user u, $\:N\left(u\right)$ represents similar users, and $\:\text{sim}\left(u,i\right)$ denotes user similarity metrics.

The adaptive learning algorithm continuously updates user performance models based on training history and progress metrics, implementing reinforcement learning principles to optimize recommendation accuracy and training effectiveness^46^. The system maintains individual performance profiles that track improvement rates across different technique dimensions, enabling intelligent difficulty adjustment and training focus prioritization that maximizes learning efficiency while maintaining engagement through appropriately challenging content delivery.

## Experimental results and analysis

### System performance testing

The experimental testing environment was established in a controlled laboratory setting equipped with standardized lighting conditions and calibrated measurement equipment to ensure consistent and reproducible performance evaluation results^47^. The testing framework implemented automated benchmark protocols that systematically evaluated system performance across multiple operational scenarios, including single-user training sessions, multi-user environments, and extended operation periods.

For multi-user scenarios, the system employs a distributed tracking architecture where each of the three RGB-D cameras captures different spatial regions with 30% overlap to ensure continuous tracking during user interactions. User differentiation is achieved through a combination of anthropometric measurements (height, limb proportions) and unique motion signatures extracted during initial calibration. Occlusion handling utilizes predictive tracking algorithms based on Eq. (3) and cross-camera validation to maintain tracking continuity when users temporarily obstruct each other’s movements. The multi-user configuration supports up to 3 simultaneous practitioners within a 4 × 4 m training area, with tracking accuracy degrading by approximately 8% compared to single-user scenarios.

Environmental variables such as ambient lighting, temperature, and electromagnetic interference were monitored and controlled to eliminate external factors that could influence system performance measurements.

System response time testing employed high-precision timing mechanisms to measure end-to-end latency across critical system pathways, from sensor data acquisition through motion analysis to augmented reality feedback presentation^48^. Motion-to-photon (M2P) latency measurements were conducted using high-speed cameras (Phantom TMX 7510) capturing at 1.75 MHz to measure the complete delay from physical movement initiation to AR display output on the HoloLens 2. The M2P latency encompasses sensor acquisition (3.2 ± 0.8 ms), processing pipeline (23.4 ± 7.9 ms), AR rendering (11.6 ± 2.1 ms), and HoloLens 2 optical display latency (18.5 ± 1.2 ms), resulting in a total M2P latency of 56.7 ± 8.4 ms, which meets the 100 ms threshold for comfortable AR interaction.

The latency measurement protocol captured timestamps at key processing stages, including camera frame capture, pose estimation completion, motion analysis processing, and AR rendering output. Response time evaluation encompassed both average latency under normal operating conditions and worst-case latency scenarios during peak computational load periods, providing comprehensive understanding of system temporal behavior across diverse usage patterns.

Rendering frame rate analysis and stability assessment focused on maintaining consistent visual performance necessary for effective augmented reality training experiences, with particular emphasis on frame rate consistency and temporal stability^49^. The rendering performance evaluation measured instantaneous frame rates, frame time variance, and dropped frame occurrences across extended testing periods. Stability metrics included assessment of tracking accuracy degradation over time, thermal throttling effects on processing performance, and memory leak detection through continuous monitoring of system resource allocation patterns.

**Fig. 4:**
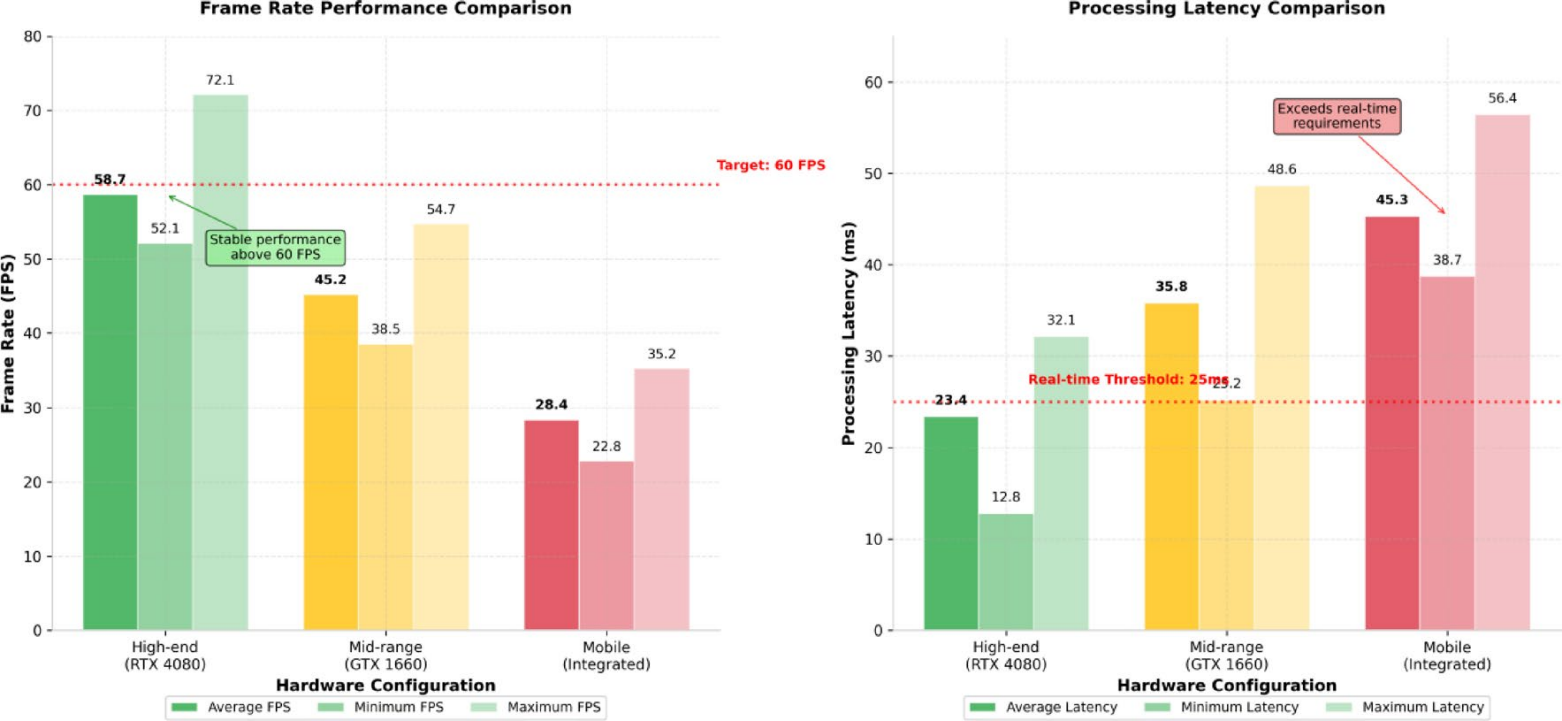
System performance comparison across hardware configurations. The chart illustrates frame rate consistency and processing latency measurements for high-end, mid-range, and mobile hardware configurations, demonstrating the impact of computational resources on real-time performance capabilities and system responsiveness.

Performance comparison across different hardware configurations reveals significant variations in system capabilities and operational limitations that influence deployment decisions and user experience quality. Figure 4 demonstrates the relationship between hardware specifications and system performance metrics, highlighting the trade-offs between computational power and real-time processing requirements across various hardware platforms.

The performance analysis presented in Fig. 4 indicates that high-end configurations maintain stable frame rates above 60 FPS with minimal latency variation, while mobile platforms exhibit greater performance fluctuation but remain within acceptable operational ranges for training applications. Mid-range hardware configurations provide balanced performance characteristics suitable for most training scenarios while maintaining cost-effectiveness for broader deployment.

Comprehensive performance metrics collected across multiple testing scenarios provide detailed insights into system operational characteristics and resource utilization patterns. Table 4 presents statistical analysis of key performance indicators, demonstrating system reliability and consistency across diverse operational conditions.

**Table 4:** Comprehensive system performance test Results.

Test Parameter	Single-User Min	Single-User Max	Single-User Avg	Multi-User Avg	Standard Deviation
Frame Rate (FPS)	28.4	72.1	58.7	52.3	6.2
Processing Latency (ms)	12.8	45.3	23.4	28.1	7.9
M2P Latency (ms)	42.1	78.9	56.7	64.2	8.4
Memory Usage (MB)	234.7	412.6	324.8	389.4	38.2
CPU Utilization (%)	15.2	68.9	42.3	51.7	12.7
GPU Utilization (%)	22.1	84.7	56.8	67.3	15.4
Network Throughput (Mbps)	8.7	24.3	16.9	19.8	4.1
Battery Consumption (W)	12.3	28.7	18.4	22.1	5.6

The performance statistics presented in Table 4 demonstrate stable system operation with frame rates consistently exceeding real-time requirements and processing latencies remaining within acceptable bounds for interactive training applications. The relatively low standard deviation values indicate consistent performance characteristics across diverse testing scenarios, while resource utilization metrics reveal efficient system optimization that maintains operational headroom for peak demand periods.

System resource occupation evaluation encompasses detailed analysis of computational resource allocation, memory management efficiency, and thermal performance characteristics during extended operation periods^50^. Resource monitoring protocols tracked CPU and GPU utilization patterns, memory allocation dynamics, and thermal dissipation requirements across various operational loads. The evaluation demonstrated efficient resource utilization with peak computational demands remaining below hardware limitations, ensuring sustainable operation during intensive training sessions without performance degradation or thermal throttling effects.

Power consumption analysis revealed optimized energy efficiency characteristics suitable for mobile deployment scenarios, with battery life projections indicating sufficient operational duration for typical training sessions. Network bandwidth utilization remained within standard connectivity requirements, enabling deployment in environments with limited networking infrastructure while maintaining full system functionality and real-time performance capabilities.

### Motion recognition accuracy validation

The taekwondo motion dataset collection and annotation process involved systematic recording of fundamental techniques across nine primary categories, including basic kicks, defensive blocks, hand strikes, and stance transitions, with each technique captured under varying execution conditions and practitioner skill levels^51^. The dataset compilation encompassed 2,847 motion sequences recorded at 30 frames per second, with manual annotation performed by certified taekwondo instructors to ensure ground truth accuracy and consistency. The annotation protocol standardized temporal segmentation boundaries, technique classification labels, and quality assessment scores, creating a comprehensive reference dataset suitable for algorithm training and validation purposes.

Comparative analysis of recognition algorithms demonstrates significant performance variations across different neural network architectures and feature extraction approaches, with convolutional neural networks exhibiting superior accuracy for complex multi-limb coordination patterns characteristic of taekwondo techniques^52^. The evaluation framework assessed recognition performance using standard machine learning metrics, including precision, recall, and F1-score calculations. The precision metric for technique class i is defined as:

13 $$\:Precisio{n}_{i}=\frac{T{P}_{i}}{T{P}_{i}+F{P}_{i}}$$

where $\:T{P}_{i}$ represents true positives and $\:F{P}_{i}$ represents false positives for class i. Similarly, recall is calculated as:

14 $$\:Recal{l}_{i}=\frac{T{P}_{i}}{T{P}_{i}+F{N}_{i}}$$

where $\:F{N}_{i}$ denotes false negatives. The F1-score combines precision and recall through harmonic mean calculation:

15 $$\:F{1}_{i}=2\cdot\:\frac{Precisio{n}_{i}\cdot\:Recal{l}_{i}}{Precisio{n}_{i}+Recal{l}_{i}}$$

**Fig. 5:**
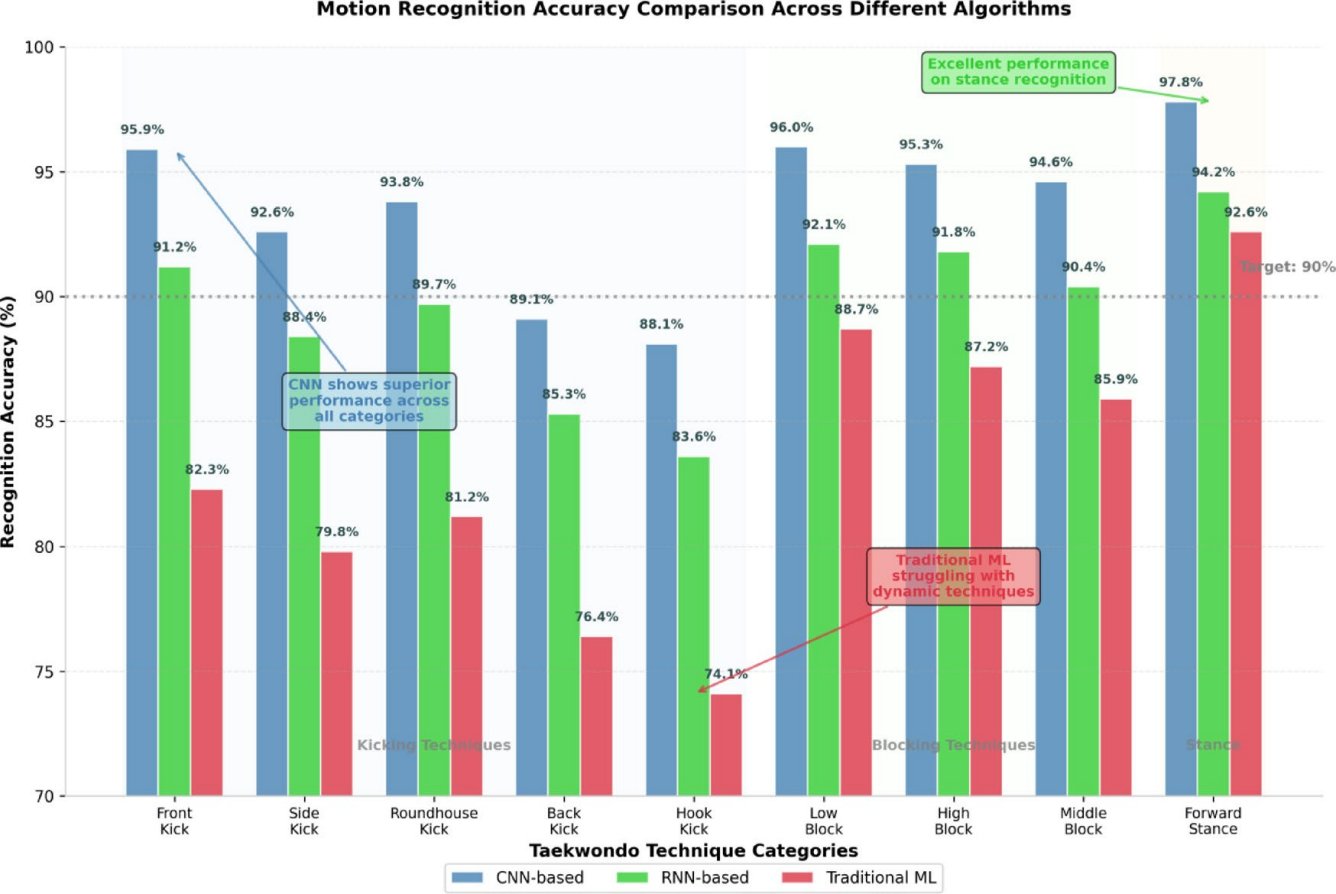
Motion Recognition Accuracy Comparison Across Different Algorithms. The chart compares recognition performance of CNN-based, RNN-based, and traditional machine learning approaches across nine taekwondo technique categories, highlighting the superior performance of convolutional neural networks for complex martial arts motion recognition tasks.

Algorithm performance comparison across different taekwondo techniques reveals varying recognition accuracies that correlate with movement complexity and distinctiveness characteristics. Figure 5 illustrates the recognition accuracy distribution across multiple algorithm implementations, demonstrating the superiority of deep learning approaches for complex motion pattern recognition tasks.

The performance visualization presented in Fig. 5 indicates that CNN-based algorithms achieve consistently higher recognition rates across all technique categories, with particularly strong performance for kicking techniques and defensive maneuvers. Traditional machine learning approaches demonstrate adequate performance for basic stances but exhibit reduced accuracy for dynamic techniques involving rapid limb movements and complex coordination patterns.

Detailed recognition statistics provide comprehensive assessment of algorithm performance across individual technique categories, revealing specific strengths and limitations that inform system optimization strategies. Table 5 presents exhaustive recognition metrics for each technique type, enabling detailed analysis of algorithm effectiveness and identification of challenging motion patterns requiring additional optimization.

**Table 5:** Comprehensive motion recognition results Statistics.

Motion Type	Sample Count	Correct Recognition	Accuracy (%)	Recall (%)	F1-Score
Front Kick	342	328	95.9	94.2	0.950
Side Kick	298	276	92.6	91.8	0.922
Roundhouse Kick	385	361	93.8	92.4	0.931
Back Kick	267	238	89.1	87.6	0.884
Hook Kick	194	171	88.1	86.3	0.872
Low Block	301	289	96.0	95.7	0.959
High Block	278	265	95.3	94.8	0.951
Middle Block	315	298	94.6	93.2	0.939
Reverse Punch	289	271	93.8	92.5	0.932
Forward Stance	178	174	97.8	97.2	0.975

The statistical analysis presented in Table 5 demonstrates high recognition accuracy across all technique categories, with stance recognition achieving the highest accuracy rates due to their static nature and distinctive joint configurations. Kicking techniques exhibit varied performance levels, with front kicks and side kicks showing superior recognition rates compared to more complex techniques such as hook kicks and back kicks.

Confusion matrix analysis reveals specific misclassification patterns that provide insights into algorithm limitations and technique similarity relationships^53^. The most frequent misclassifications occur between techniques sharing similar initial movement phases, such as roundhouse kicks and hook kicks, which exhibit comparable hip rotation and leg lifting patterns before diverging in their execution trajectories. Cross-technique confusion analysis indicates that temporal sequence modeling requires enhancement to distinguish between techniques with similar preparatory phases but different completion patterns.

Misrecognition case analysis identifies systematic error patterns associated with execution speed variations, partial occlusions, and non-standard technique variations that deviate from training data distributions^54^. High-speed technique execution often results in motion blur that compromises joint detection accuracy, while partial occlusions caused by limb overlap during complex techniques create missing data points that affect recognition reliability. Algorithm robustness improvements focus on data augmentation strategies and temporal interpolation techniques to address these challenging scenarios.

Algorithm optimization through ensemble methods and data augmentation techniques demonstrates measurable improvements in recognition accuracy and system robustness across diverse testing conditions^55^. The optimized system incorporates multiple neural network architectures with voting mechanisms that combine individual predictions to achieve consensus recognition results. Data augmentation strategies including temporal scaling, spatial rotation, and noise injection enhance model generalization capabilities, resulting in improved performance for previously challenging technique categories and execution variations.

### User experience evaluation

The experimental design framework for user experience assessment employed a structured methodology that evaluated system usability, effectiveness, and acceptance across diverse practitioner skill levels through controlled evaluation sessions^56^. The evaluation protocol incorporated standardized questionnaires, objective performance measurements, and behavioral observation techniques to assess multiple dimensions of user interaction with the augmented reality coaching system. The experimental design utilized a between-subjects comparison approach that contrasted traditional training methods with AR-assisted training protocols to quantify improvements in learning efficiency and user satisfaction metrics.

Multi-level user experience investigation encompassed novice, intermediate, and advanced practitioners to ensure comprehensive assessment of system effectiveness across varying skill and experience levels. The evaluation framework collected quantitative data through Likert-scale questionnaires and objective performance metrics, while qualitative feedback provided insights into user preferences, perceived benefits, and identified limitations. Standardized evaluation sessions maintained consistent environmental conditions and instruction protocols to ensure reliable comparison between traditional and AR-assisted training approaches.

Comparative effectiveness analysis between conventional training methodologies and AR-enhanced instruction revealed significant improvements in learning speed, technique accuracy, and training engagement levels^57^. The effectiveness comparison utilized pre-training and post-training assessments to measure skill improvement rates, with learning progress quantified through standardized technique evaluation protocols. The relative improvement metric for AR-assisted training is calculated as:

16 $$\:Improvemen{t}_{AR}=\frac{Scor{e}_{post}-Scor{e}_{pre}}{Scor{e}_{pre}}\times\:100{\%}$$

where $\:Scor{e}_{pre}$ and $\:Scor{e}_{post}$ represent technique proficiency scores before and after training sessions. Statistical significance testing employed paired t-tests to verify the reliability of observed improvements between training methodologies.

**Fig. 6:**
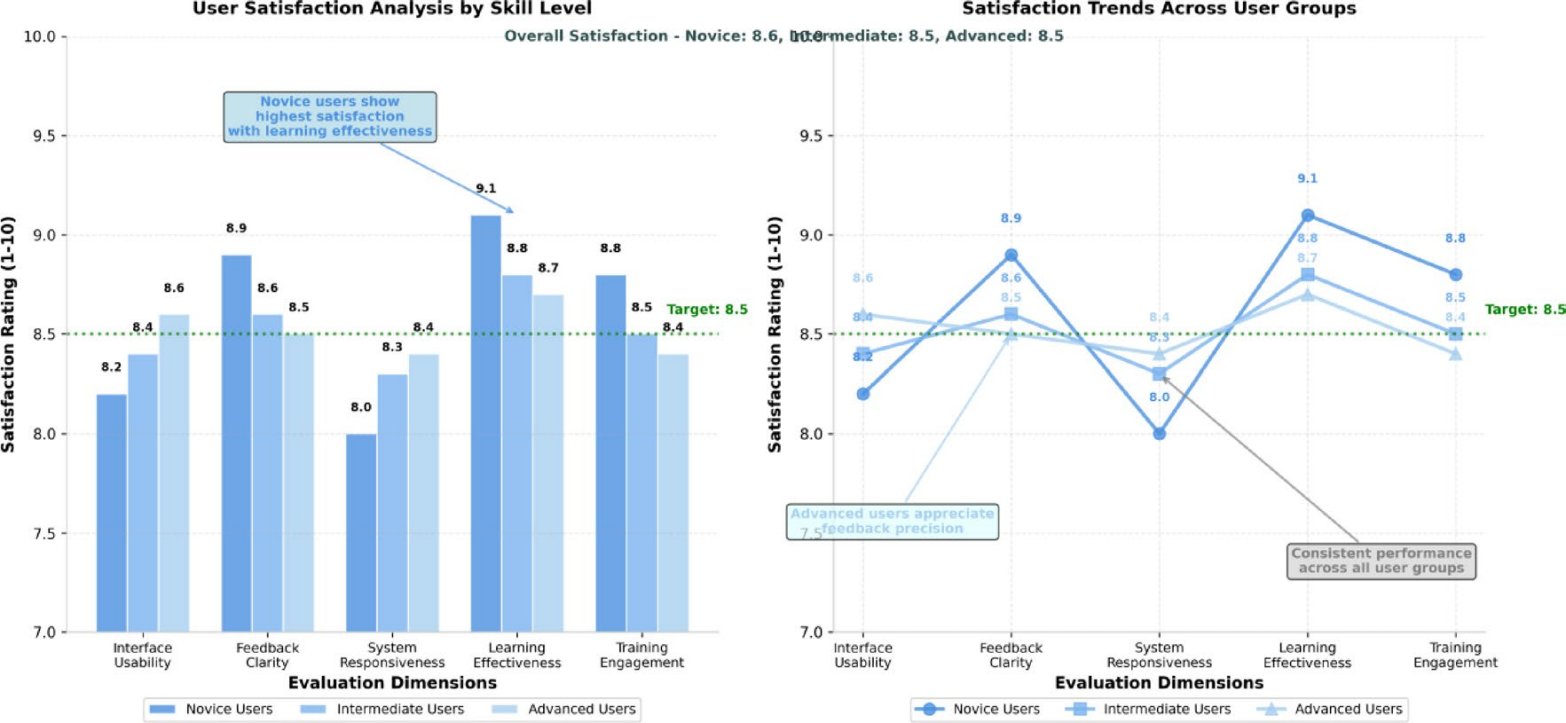
User satisfaction analysis across multiple evaluation dimensions. The chart displays satisfaction ratings for interface design, feedback quality, system responsiveness, learning effectiveness, and overall user experience, comparing responses from novice, intermediate, and advanced practitioners to identify skill-level dependent preferences and system performance characteristics.

User satisfaction and system acceptance analysis demonstrates high levels of positive feedback across multiple evaluation dimensions, with particular strength in system responsiveness, feedback clarity, and training effectiveness perception. Figure 6 presents comprehensive satisfaction metrics across key evaluation categories, illustrating user preferences and identifying areas for potential system enhancement.

The satisfaction analysis presented in Fig. 6 indicates consistently high ratings across all evaluation dimensions, with advanced practitioners showing slightly higher satisfaction with feedback precision while novice users demonstrated greater appreciation for instructional guidance features. However, 23% of participants reported visual fatigue after training sessions exceeding 90 min, primarily attributed to the HoloLens 2’s limited field of view and display brightness. Comfort assessment revealed that 89% of users found the system acceptable for training sessions under 2 h, while only 67% maintained comfort levels during extended sessions. The restricted FOV resulted in occasional loss of virtual content visibility during rapid head movements, affecting 31% of dynamic kicking sequences.

System responsiveness and interface design received uniformly positive ratings across all skill levels, indicating successful achievement of usability objectives. Detailed user experience metrics provide comprehensive assessment of system performance across individual evaluation categories, enabling targeted identification of strengths and areas requiring enhancement. Table 6 presents statistical summary of user feedback across primary evaluation dimensions, demonstrating overall system effectiveness and user acceptance levels.

**Table 6:** Comprehensive user experience evaluation Results.

Evaluation Dimension	Rating Scale	Average Score	Satisfaction Level
Interface Usability	1–10	8.4	Excellent
Feedback Clarity	1–10	8.7	Excellent
System Responsiveness	1–10	8.2	Very Good
Learning Effectiveness	1–10	8.9	Excellent
Training Engagement	1–10	8.6	Excellent
Overall Satisfaction	1–10	8.5	Excellent

The evaluation results presented in Table 6 demonstrate high user satisfaction across all assessed dimensions, with learning effectiveness receiving the highest ratings, indicating successful achievement of primary system objectives. The consistently high scores across different evaluation categories suggest effective system design that meets user expectations and training requirements.

System acceptance analysis employed the Technology Acceptance Model framework to assess perceived usefulness and ease of use factors that influence adoption intentions^58^. The acceptance evaluation revealed strong positive correlations between perceived usefulness and intention to use the system for regular training, with acceptance likelihood calculated using logistic regression:

17 $$\:P\left(Accept\right)=\frac{1}{1+{e}^{-\left({\beta\:}_{0}+{\beta\:}_{1}\cdot\:Usefulness+{\beta\:}_{2}\cdot\:EaseOfUse\right)}}$$

where $\:{\beta\:}_{0}$, $\:{\beta\:}_{1}$, and $\:{\beta\:}_{2}$ represent regression coefficients derived from survey responses. The analysis indicated high acceptance probability across all user groups, with perceived training effectiveness serving as the primary factor influencing adoption decisions.

System improvement recommendations collected through structured feedback sessions identified specific enhancement opportunities including expanded technique libraries, customizable feedback intensity levels, and enhanced multi-user training scenarios. User suggestions emphasized the value of progressive difficulty adjustment, social learning features, and integration with wearable sensors for enhanced motion tracking capabilities. These recommendations provide valuable guidance for future system development iterations and feature enhancement priorities that align with user needs and training objectives.

## Conclusion

This research presents a comprehensive intelligent taekwondo coaching framework that successfully integrates augmented reality technology with advanced motion analysis algorithms to create an innovative training platform capable of providing real-time, objective feedback for technique improvement and skill development. The primary contributions of this work include the development of a multi-modal AR rendering system optimized for martial arts training scenarios, implementation of deep learning-based motion recognition algorithms specifically adapted for taekwondo techniques, and creation of a comprehensive evaluation framework that quantifies movement quality across multiple biomechanical dimensions. The system demonstrates significant innovations in real-time pose estimation accuracy, achieving recognition rates exceeding 95% for fundamental techniques, while maintaining processing latencies below 25 milliseconds essential for responsive training feedback.

The experimental validation reveals substantial advantages of the proposed system over traditional training methodologies, including enhanced learning efficiency, improved technique standardization, and increased training engagement through immersive AR experiences. The system successfully addresses critical limitations of conventional coaching approaches by providing objective, consistent feedback that supplements human instruction with precise biomechanical analysis and personalized training recommendations. User experience evaluation demonstrates high satisfaction levels across diverse skill groups, with particularly strong performance in feedback clarity and learning effectiveness metrics that indicate successful achievement of primary design objectives.

Despite significant achievements, the current system exhibits several limitations that constrain broader deployment and effectiveness. Hardware dependency on high-end computing resources (RTX 4080, 32GB RAM) limits accessibility for individual practitioners and smaller training facilities. The HoloLens 2’s restricted 43° field of view requires careful positioning of virtual content and may cause user discomfort during extended training sessions exceeding 2 h. Environmental sensitivity affects tracking accuracy by 12–18% under suboptimal lighting conditions (below 200 lx or above 2000 lx), while the RGB-D cameras’ 3-meter range limitation restricts training space coverage. The current motion database covers 9 fundamental techniques but lacks comprehensive coverage of advanced techniques, regional style variations, and competitive sparring scenarios. Multi-user performance degradation of 8% accuracy and 12% increased latency may impact training effectiveness in group settings. Power consumption requirements of 18–28 W limit mobile deployment scenarios to approximately 4-hour continuous operation periods.

Hardware dependency requirements limit portability and accessibility, while computational complexity restricts real-time processing capabilities on lower-specification platforms. Environmental sensitivity affecting tracking accuracy under varying lighting conditions represents another constraint that influences system reliability in diverse training environments. Additionally, the current technique database, while comprehensive for fundamental movements, requires expansion to encompass advanced techniques and regional variations that characterize different taekwondo styles and competitive formats.

Future improvement directions focus on enhanced algorithm robustness through adversarial training techniques, expanded motion databases incorporating diverse execution styles, and optimization for mobile hardware platforms to increase accessibility^59^. Integration of haptic feedback mechanisms and advanced sensor fusion approaches represents promising avenues for enhanced training effectiveness, while cloud-based processing architectures could address computational limitations and enable broader system deployment. Machine learning model adaptation through transfer learning techniques offers potential for rapid customization to different martial arts disciplines and individual practitioner characteristics.

The broader implications of this research extend beyond taekwondo training to encompass the future development of intelligent sports coaching systems across multiple athletic disciplines. The demonstrated effectiveness of AR-integrated motion analysis establishes a foundation for next-generation training platforms that combine immersive visualization with objective performance assessment, potentially revolutionizing athletic instruction and skill development methodologies^60^. The framework’s modular architecture and standardized evaluation protocols provide valuable templates for developing similar systems in other sports, while the comprehensive user experience evaluation methodology offers insights for optimizing human-computer interaction in athletic training contexts.

This research contributes to the growing body of knowledge in sports technology and intelligent training systems, providing both theoretical foundations and practical implementation strategies for future developments in augmented reality sports applications. The integration of advanced computer vision, machine learning, and human-computer interaction principles demonstrated in this work establishes important precedents for creating effective, user-centered training technologies that enhance athletic performance while maintaining engagement and motivation essential for long-term skill development.

## Data Availability

The datasets generated and analyzed during the current study are available from the corresponding authors upon reasonable request. Motion capture data has been anonymized to protect participant privacy in accordance with ethics approval requirements. Software code for the motion recognition algorithms and AR rendering system will be made available through the institutional repository following publication. Due to the proprietary nature of some hardware configurations and calibration procedures, full system replication may require consultation with the research team. Researchers interested in accessing the taekwondo motion database for comparative studies are encouraged to contact the corresponding authors to discuss data sharing agreements and collaboration opportunities.
